# *Aspergillus oryzae* AoSO Is a Novel Component of Stress Granules upon Heat Stress in Filamentous Fungi

**DOI:** 10.1371/journal.pone.0072209

**Published:** 2013-08-21

**Authors:** Hsiang-Ting Huang, Jun-ichi Maruyama, Katsuhiko Kitamoto

**Affiliations:** Department of Biotechnology, The University of Tokyo, Tokyo, Japan; Universidade de Sao Paulo, Brazil

## Abstract

Stress granules are a type of cytoplasmic messenger ribonucleoprotein (mRNP) granule formed in response to the inhibition of translation initiation, which typically occurs when cells are exposed to stress. Stress granules are conserved in eukaryotes; however, in filamentous fungi, including *Aspergillus oryzae*, stress granules have not yet been defined. For this reason, here we investigated the formation and localization of stress granules in *A. oryzae* cells exposed to various stresses using an EGFP fusion protein of AoPab1, a homolog of *Saccharomyces cerevisiae* Pab1p, as a stress granule marker. Localization analysis showed that AoPab1 was evenly distributed throughout the cytoplasm under normal growth conditions, and accumulated as cytoplasmic foci mainly at the hyphal tip in response to stress. AoSO, a homolog of *Neurospora crassa* SO, which is necessary for hyphal fusion, colocalized with stress granules in cells exposed to heat stress. The formation of cytoplasmic foci of AoSO was blocked by treatment with cycloheximide, a known inhibitor of stress granule formation. Deletion of the *Aoso* gene had effects on the formation and localization of stress granules in response to heat stress. Our results suggest that AoSO is a novel component of stress granules specific to filamentous fungi.

The authors would specially like to thank Hiroyuki Nakano and Kei Saeki for generously providing experimental and insightful opinions.

## Introduction

The ability to sense environmental stimuli, including stress, activate signal transduction, and mount appropriate acute and adaptive responses is crucial for eukaryotic cell survival. Adaptation is achieved through the regulation of gene expression. Traditionally, transcriptional regulation has been regarded as the major determinant of gene expression. However, accumulating evidence indicates that posttranscriptional modulation of mRNA stability and translation plays a key role in the control of gene expression and provides greater plasticity, allowing cells to immediately adjust protein synthesis in response to changes in the environment [1]. Recent studies have demonstrated that one aspect of this modulation involves the remodeling of mRNAs translated from polysomes into non-translating messenger ribonucleoproteins (mRNPs), which accumulate in discrete cytoplasmic foci known as stress granules and processing bodies (P-bodies) [2]–[8].

Environmental stress response mechanisms in eukaryotic cells are characterized by global translational inhibition, which inhibits protein synthesis to conserve anabolic energy and also involves the reconfiguration of gene expression to effectively manage stress conditions. Global translational arrest is initiated by the activation of several stress-responsive serine/threonine kinases, including general control non-derepressible-2 (GCN2), dsRNA-dependent protein kinase R (PKR), heme-regulated inhibitor kinase (HRI), and PKR-like ER kinase (PERK), which phosphorylate the translation initiation factor eIF2α [9]. eIF2α is a subunit of eIF2 (together with eIF2β and eIF2γ), which is part of a ternary complex, consisting of eIF2, GTP, and methionyl-initiator tRNA (Met-tRNAi^ Met^), that delivers initiator tRNA to the 40S ribosome [1]. During translation initiation, GTP is hydrolyzed to GDP, generating eIF2-GDP, which needs to be recharged to eIF2-GTP by a guanine nucleotide exchange factor following each round of initiation. Phosphorylation of eIF2 by eIF2α kinases inhibits the formation of eIF2-GTP, thereby reducing the level of the ternary complex, which ultimately limits translation initiation [1]. The stalled preinitiation complexes, together with their associated mRNAs, are routed into stress granules. However, a subset of mRNAs required for cell survival under stress conditions are not delivered to stress granules but stabilized and preferentially translated in the cytoplasm [10]–[13]. As stress granules are formed in response to stress, they are generally not observable under normal growth. Although the composition of stress granules is dynamic and dependent on the type of stress and cell species, they typically contain 40S ribosomal subunits, translation initiation factors (eIF4G, eIF4E, eIF4A, eIF4B, eIF3, and eIF2), and proteins involved in the regulation of mRNA function [5], [14]. The function of stress granules is not fully understood; however, they have been implicated in the posttranscriptional regulation processes, such as mRNA translational repression and storage, and cellular signal transduction [4], [5], [15], [16].

Eukaryotic mRNA degradation is generally initiated by the deadenylation of poly(A) tails, which triggers either decapping and 5′ to 3′ exonucleolysis or exosome-dependent 3′ to 5′ degradation [17]–[20]. As the inhibition of translation initiation increases the rate of deadenylation and decapping [21]–[24], the rate of mRNA degradation and translation initiation are often inversely related [7], [25]. The cellular components involved in mRNA decapping and degradation, such as decapping enzyme complex Dcp1p/Dcp2p, the activators of decapping and/or repressors of translation Dhh1p/RCK/p54, Lsm1–7p complex, Edc3p, Pat1p and the 5′ to 3′ exonuclease Xrn1p, often accumulate in P-bodies as core components [26]–[28]. In addition, proteins functioning in different posttranscriptional processes, including microRNA (miRNA)-mediated silencing, AU-rich element (ARE)-mRNA decay, mRNA surveillance (nonsense-mediated decay, NMD), and mRNA storage [2], [27], [29], have also been reported to localize in P-bodies under certain conditions.

The mycelia of filamentous fungi consist of a network of interconnected hyphae, which are compartmentalized by septa. Septa contain a central pore that allows the movement of cytoplasm and organelles between adjacent hyphae for direct communication and coordination [30]–[32]. However, mycelia with cytoplasmic continuity are susceptible to catastrophic failure due to cytoplasmic loss when individual hyphae are injured. Fungi defend against such loss by the rapid occlusion of septal pores in response to hyphal damage and stressful environmental conditions [32]–[34]. The *Neurospora crassa* SO (SOFT) protein, and its *Sordaria macrospora* homolog, Pro40, were shown to be essential for hyphal fusion [35]–[37] and sexual development [38], respectively. Subcellular localization studies revealed that SO is evenly distributed throughout the cytoplasm under normal growth conditions, but accumulates at the septal pore in injured, aging, and dying hyphae [35]. We have also shown previously that an *Aspergillus oryzae* homolog of SO, AoSO, accumulates at the septal pore when cells are exposed to various stresses [39]. The stress-induced accumulation behavior of AoSO suggests that it may interact with stress granules.

Stress granules are conserved in eukaryotes and have been studied extensively in yeast and mammalian cells. However, the existence and function of stress granules in filamentous fungi, including *A. oryzae*, have not yet to be defined. For this reason, in the present work, we investigated the formation and localization of stress granules in *A. oryzae* cells exposed to various stresses using an EGFP fusion protein of AoPab1, a homolog of *Saccharomyces cerevisiae* Pab1p, as a stress granule marker. Moreover, subcellular localization studies showed that AoSO protein accumulated at the septal pore and also in cytoplasmic foci at the hyphal tip when cells were exposed to heat stress. The cytoplasmic AoSO foci at the hyphal tip were cycloheximide sensitive and colocalized with stress granules. Deletion of the *Aoso* gene had effects on the formation and localization of stress granules in the *Aoso* disruptant. Together, the results presented in this study reveal that AoSO has a novel function in stress granules in *A. oryzae*.

## Materials and Methods

### Plasmid construction

*Escherichia coli* DH5α was used for DNA manipulations. Plasmids for the expression of EGFP-fused proteins were constructed using the MultiSite Gateway system (Invitrogen, Carlsbad, CA, USA), as previously described [40]. ORF regions of *Aopab1* (AO090003000927), *Aopub1* (AO090001000353), and *Aodcp2* (AO090120000363) genes were amplified by PCR using the genomic DNA of *A. oryzae* wild-type strain RIB40 [41] as a template, and then cloned into pDONR221 by the BP clonase reaction, generating center entry clones pgEpab1, pgEpub1, and pgEdcp2, respectively. The center entry clones were individually mixed with 5′ entry clone pg5'PaB, 3′ entry clone pg3'E, and destination vector pgDN [40] for the LR clonase reaction, generating plasmids pgDPapab1E, pgDPapub1E, and pgDPadcp2E, respectively. For the expression of AoPab1-mDsRed, pgEpab1 was mixed with 5′ entry clone pg5'PaB, 3′ entry clone pg3'DRM-CF, and destination vector pgDSO [42] for the LR clonase reaction, generating plasmid pgCPapab1DR.

To generate the template DNA fragment for disruption of *Aopub1*, the 5′- and 3′-flanking regions of *Aopub1* were amplified by PCR using genomic DNA of wild-type strain RIB40 as a template, and introduced into plasmids pDONR P4-P1R and pDONR P2R-P3 by the BP clonase reaction, generating the 5′ entry clone pg5Pub1up and 3′ entry clone pg3Pub1dw, respectively. Plasmids pg5Pub1up and pg3Pub1dw were mixed with center entry clone pgEpG and destination vector pDEST R4-R3 for the LR clonase reaction to generate plasmid pgdPub1.

### *A. oryzae* strains and transformation

The *A*. *oryzae* strains used in this study are listed in Table 1. Transformation of *A. oryzae* was carried out according to the standard method described previously [43], [44]. To generate EGFP-expressing strains, plasmids pgPapab1E and pgPapub1E were introduced into strain NSRKu70-1-1A [42]; while plasmid pgPadcp2E was introduced into strain NS4 [45]. The resulting transformants were selected using Czapek-Dox (CD) medium (0.3% NaNO_3_, 0.2% KCl, 0.1% KH_2_PO_4_, 0.05% MgSO_4_·7H_2_O, 0.002% FeSO_4_·7H_2_O, and 2% glucose, pH 5.5) supplemented with 0.15% methionine (CD + Met).

**Table 1:** *A. oryzae* strains used in this study.

Strain	Host	Genotype	Reference
RIB40		Wild-type	41
NSRKu70-1-1A	NSRKu-70-1-1	*niaD* ^−^ *sC* ^−^ *adeA* ^−^ Δ*argB*::*adeA* ^−^ Δ*ku70*::*argB adeA*	42
NS4	niaD300	*niaD* ^−^ *sC* ^−^	45
NSPlD1	NSlD	*niaD* ^−^ *sC* ^−^ *adeA* ^−^ Δ*argB*::*adeA* ^−^ Δ*ligD*::*argB* Δ*pyrG*::*adeA*	46
NSlD1	NSPlD1	*niaD* ^−^ *sC* ^−^ *adeA* ^−^ Δ*argB*::*adeA* ^−^ Δ*ligD*::*argB* Δ*pyrG*::*adeA pyrG*	46
NSK-ASG1	NSK-ΔSO11	*niaD* ^−^ *sC* ^−^ *adeA* ^−^ Δ*argB*::*adeA* ^−^ Δ*ku70*::*argB* Δ*Aoso*::*adeA* pgPaBSG [P*amyB*::*Aoso-egfp*::T*amyB*::*niaD*]	39
SK-Pab1E	NSRKu70-1-1A	*niaD* ^−^ sC^−^ *adeA* ^−^ Δ*argB::adeA* ^−^ Δ*ku70*::*argB adeA* pgDPapab1E [P*amyB*::*Aopab1*-*egfp*::T*amyB*::*niaD*]	This study
SK-Pub1E	NSRKu70-1-1A	*niaD* ^−^ sC^−^ *adeA* ^−^ Δ*argB::adeA* ^−^ Δ*ku70*::*argB adeA* pgDPapub1E [P*amyB*::*Aopub1*-*egfp*::T*amyB*::*niaD*]	This study
SK-E	NSRKu70-1-1A	*niaD* ^−^ *sC* ^−^ *adeA* ^−^ Δ*argB*::*adeA* ^−^ Δ*ku70*::*argB adeA* pgPaE [P*amyB*:: *egfp*::T*amyB*::*niaD*]	This study
S-Dcp2E	NS4	*niaD* ^−^ *sC* ^−^ pgDPadcp2E [P*amyB*::*Aodcp2*-*egfp*::T*amyB*::*niaD*]	This study
SK-ΔSO11-Pab1E	NSK-ΔSO11	*niaD* ^−^ *sC* ^−^ *adeA* ^−^ Δ*argB*::*adeA* ^−^ Δ*ku70*::*argB* Δ*Aoso*::*adeA* pgDPapab1E [P*amyB*::*Aopab1*-*egfp*::T*amyB*::*niaD*]	This study
Dcp2E-Pab1DR	S-Dcp2E	*niaD* ^−^ *sC* ^−^ pgDPadcp2E (P*amyB*::*Aodcp2*-*egfp*::T*amyB*::*niaD*) pgCPab1DR [P*amyB*::*Aopab1*-*mDsRed*::T*amyB*::*sC*]	This study
SK-ΔSO11-Pab1E	NSK-ΔSO11	*niaD* ^−^ *sC* ^−^ *adeA* ^−^ Δ*argB*::*adeA* ^−^ Δ*ku70*::*argB* Δ*Aoso*::*adeA* pgDPapab1E [P*amyB*::*Aopab1*-*egfp*::T*amyB*::*niaD*]	This study
NSK-ASG1-Pab1DR	NSK-ASG1	*niaD* ^−^ *sC* ^−^ *adeA-* Δ*argB*::*adeA-* Δ*ku70*::*argB* Δ*Aoso*::*adeA* pgPaBSG [P*amyB*::*Aoso-egfp*::T*amyB*::*niaD*]	This study
		pgCPab1DR [P*amyB*::*Aopab1*-*mDsRed*::T*amyB*::*sC*]	
NSK-ASG1-Pab13HA	NSK-ASG1	*niaD* ^−^ *sC* ^−^ *adeA-* Δ*argB*::*adeA-* Δ*ku70*::*argB* Δ*Aoso*::*adeA* pgPaBSG [P*amyB*::*Aoso-egfp*::T*amyB*::*niaD*]	This study
		pgCPab13HA [P*amyB*::*Aopab1*-*3HA*::T*amyB*::*sC*]	
NSK-EGFP-Pab13HA	SK-E	*niaD* ^−^ *sC* ^−^ *adeA* ^−^ Δ*argB*::*adeA* ^−^ Δ*ku70*::*argB adeA* pgPaE [P*amyB*:: *egfp*::T*amyB*::*niaD*]	This study
		pgCPab13HA [P*amyB*::*Aopab1*-*3HA*::T*amyB*::*sC*]	
NSlD-ΔPub1	NSPlD1	*niaD* ^−^ *sC* ^−^ *adeA* ^−^Δ*argB*::*adeA* ^−^ Δ*ligD*::*argB* Δ*pyrG*::*adeA* Δ*Aopub1*::*pyrG*	This study

To generate a strain co-expressing AoDcp2-EGFP and AoPab1-mDsRed, plasmid pgCPapab1DR was introduced into strain S-Dcp2E, and positive transformants were selected on CD medium. To generate a strain co-expressing AoSO-EGFP and AoPab1-mDsRed, plasmid pgCPapab1DR was introduced into strain NSK-ASG1 [39], and positive transformants were selected on CD medium. To express AoPab1-EGFP in an *Aoso* deletion background, plasmid pgDPapab1E was introduced into Δ*Aoso* strain (NSK-SO11) [39], and positive transformants were selected on CD+Met medium.

To generate an *Aopub1* disruptant, a DNA fragment containing the 5′-flanking region of *Aopub1*, *pyrG* marker, and 3′-flanking region of *Aopub1*, was amplified by PCR from plasmid pgdPub1, purified and then introduced into *A. oryzae* strain NSPlD1 [46]. Deletion of *Aopub1* was confirmed by Southern blot analysis. Briefly, genomic DNA of the strain was digested with the restriction enzymes *Bgl*II and *Bam*HI (Takara, Otsu, Japan), and was separated in a 0.8% gel by electrophoresis. The DNA was then transferred onto a Hybond N+ membrane (GE Healthcare, Buckinghamshire, UK) and detected with specific probes using the ECL Detection kit (GE Healthcare) and a LAS-4000 miniEPUV luminescent image analyzer (Fujifilm, Tokyo, Japan). The 1.5-kb downstream flanking regions of *Aopub1* amplified from pgdPub1 were used as probes.

### Fluorescence microscopy

For microscopic examination, approximately 10^4^ conidia of cells were inoculated into 100 µl CD or CD+Met media in a glass-bottom dish (Asahi Techno Glass, Chiba, Japan), and incubated at 30°C for 18 h before being exposed to stress conditions. For temperature stress, cells were shifted from 30°C to 4°C for 30 min or to 45°C for 10 min. For glucose deprivation, cells were washed three times with CD medium without glucose, and further incubated for 10 min in CD medium without glucose. For ER, osmotic, and oxidative stresses, culture medium was removed and replaced by medium containing 10 mM DTT, 1.2 M sorbitol, or 2 mM H_2_O_2_, respectively, and cells were further incubated for 60, 30, and 30 min. Stressed cells were observed by confocal microscopy using an IX71 inverted microscope (Olympus, Tokyo, Japan) equipped with a 100× Neofluor objective lens (1.40 numerical aperture), 488-nm (Furukawa Electric, Tokyo, Japan) and 561-nm semiconductor lasers (Melles Griot, Carlsbad, CA, USA), GFP, DsRed, and DualView filters (Nippon Roper, Chiba, Japan), a CSU22 confocal scanning system (Yokogawa Electronics, Tokyo, Japan), and an Andor iXon cooled digital CCD camera (Andor Technology PLC, Belfast, UK). Images were analyzed with Andor iQ software (Andor Technology PLC) and representative images are shown.

## Results

### Stress granule formation is induced in response to stress

**Figure 1:**
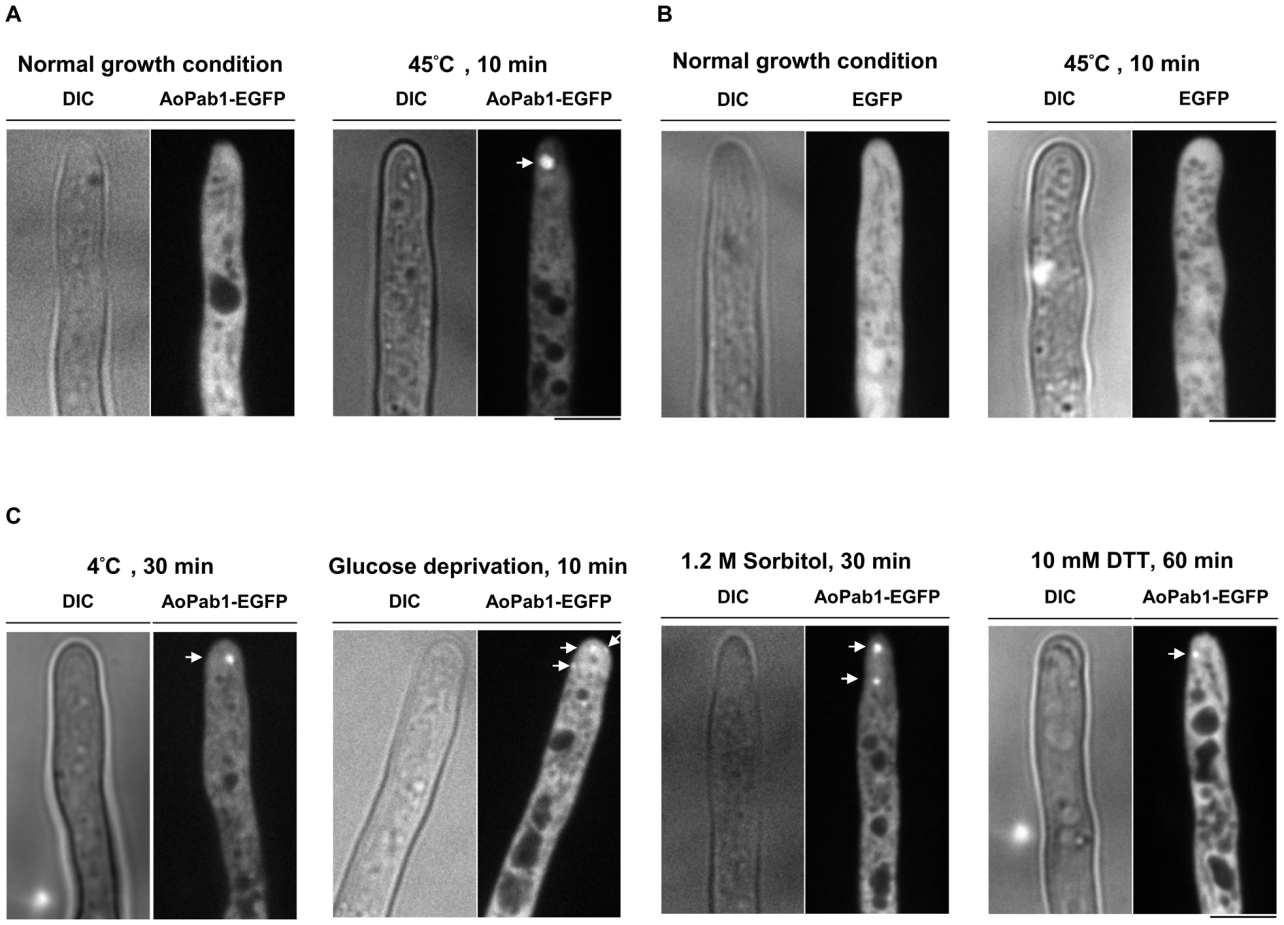
Stress-induced formation of stress granules. Approximately 10^4^ conidia of cells expressing AoPab1-EGFP were grown in CD+Met medium at 30°C for 18 h before being exposed to various types of stress. (A) Subcellular localization of AoPab1-EGFP. Accumulation of AoPab1-EGFP (indicated by the arrow) was induced when cells were exposed to 45°C for 10 min. (B) Subcellular localization of EGFP. Accumulation of EGFP was not observed in cells exposed to heat stress. (C) Accumulation of AoPab1-EGFP (indicated by the arrows) was induced in cells treated with cold stress (4°C, 30 min), glucose deprivation (10 min), osmotic stress (1.2 M sorbitol, 30 min), and ER stress (10 mM DTT, 60 min). Scale bars  =  5 µm.

**Figure 2:**
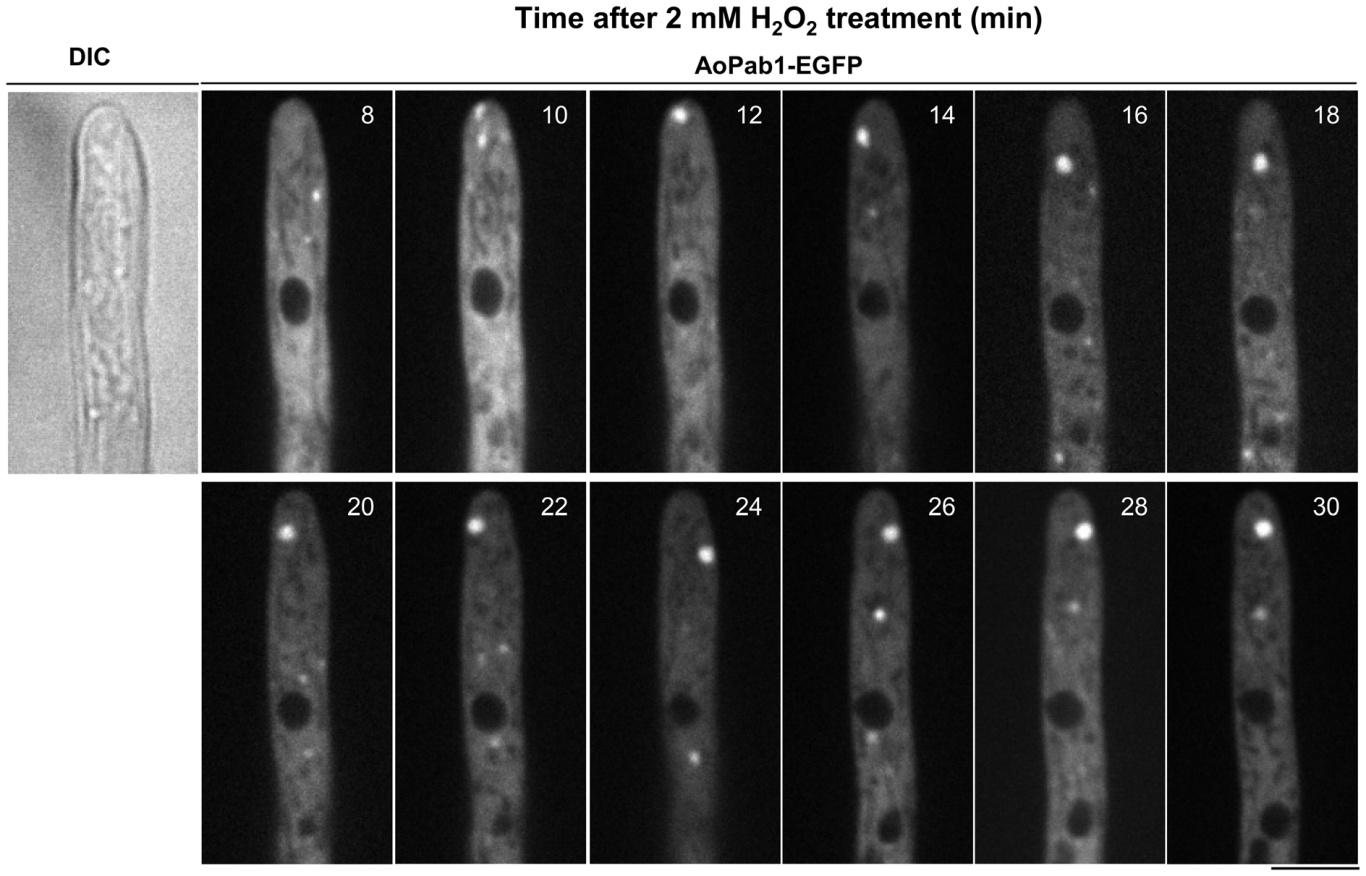
Time-lapse observation of stress granule formation upon oxidative stress. Approximately 10^4^ conidia of cells expressing AoPab1-EGFP were grown in CD+Met medium at 30°C for 18 h before being exposed to oxidative stress (2 mM H_2_O_2_). Accumulation of AoPab1-EGFP in cells was observed in a time-lapse manner. Scale bar  =  5 µm.

The polyA-binding protein Pab1p, which is involved in the translational regulation and stability of mRNAs [47], [48], is one of the most reliable and easily visualized components of stress granules in *S. cerevisiae* [49]. A homolog of the *S. cerevisiae PAB1* gene was found in the *A. oryzae* genome database (DOGAN; http://www.bio.nite.go.jp/dogan/Top) and designated as *Aopab1* (AO090003000927). To monitor stress granules by fluorescence microscopy, an AoPab1-EGFP fusion protein was expressed in strain SRK-Pab1E under control of the *amyB* promoter as a stress granule marker. Global translational arrest is a common environmental stress response in eukaryotes [50], and the inhibition of translation initiation leads to the formation of stress granules. Therefore, as a first step in characterizing stress granules in *A. oryzae*, several external stimuli were used to assess the induction of stress granules in cells (Figure 1). Under normal growth conditions, AoPab1-EGFP was dispersed throughout the cytoplasm (Figure 1A). Exposing cells to heat stress (45°C, 10 min) led to an induction of stress granules, as judged by the accumulation of AoPab1-EGFP as cytoplasmic foci (Figure 1A). No accumulation of EGFP was observed in the negative control strain expressing EGFP alone (Figure 1B). Similarly, stress granules were observed after cells were exposed to cold stress (4°C, 30 min), glucose deprivation (10 min), osmotic stress (1.2 M sorbitol, 30 min), ER stress (10 mM DTT, 60 min), and oxidative stress (2 mM H_2_O_2_, 30 min) (Figure 1C and Figure 2). In response to glucose deprivation, the accumulation of AoPab1-EGFP appeared to be short lived, as no accumulation was observed after 30 min in the majority of cells (data not shown). Small foci of AoPab1-EGFP were formed when the culture medium was replaced with medium containing 2 mM H_2_O_2_, and continued to increase in size until they fused together to form one large aggregate (Figure 2 and Video S1). Of the examined stresses, osmotic stress might be the most severe to cells, as accumulation of AoPab1-EGFP was observed less than 1 min after exposure to 1.2 M sorbitol, and a large aggregate of AoPab1-EGFP was formed shortly thereafter (data not shown). To ensure that these observations were not unique to AoPab1, we also examined the PolyA/U-binding protein AoPub1 (AO090001000353), which is another well-known component of stress granules, under the same conditions. Similar results were consistently observed in cells expressing AoPub1-EGFP (see the last section of Results).

### Stress granules colocalize with P-bodies in response to heat stress

**Figure 3:**
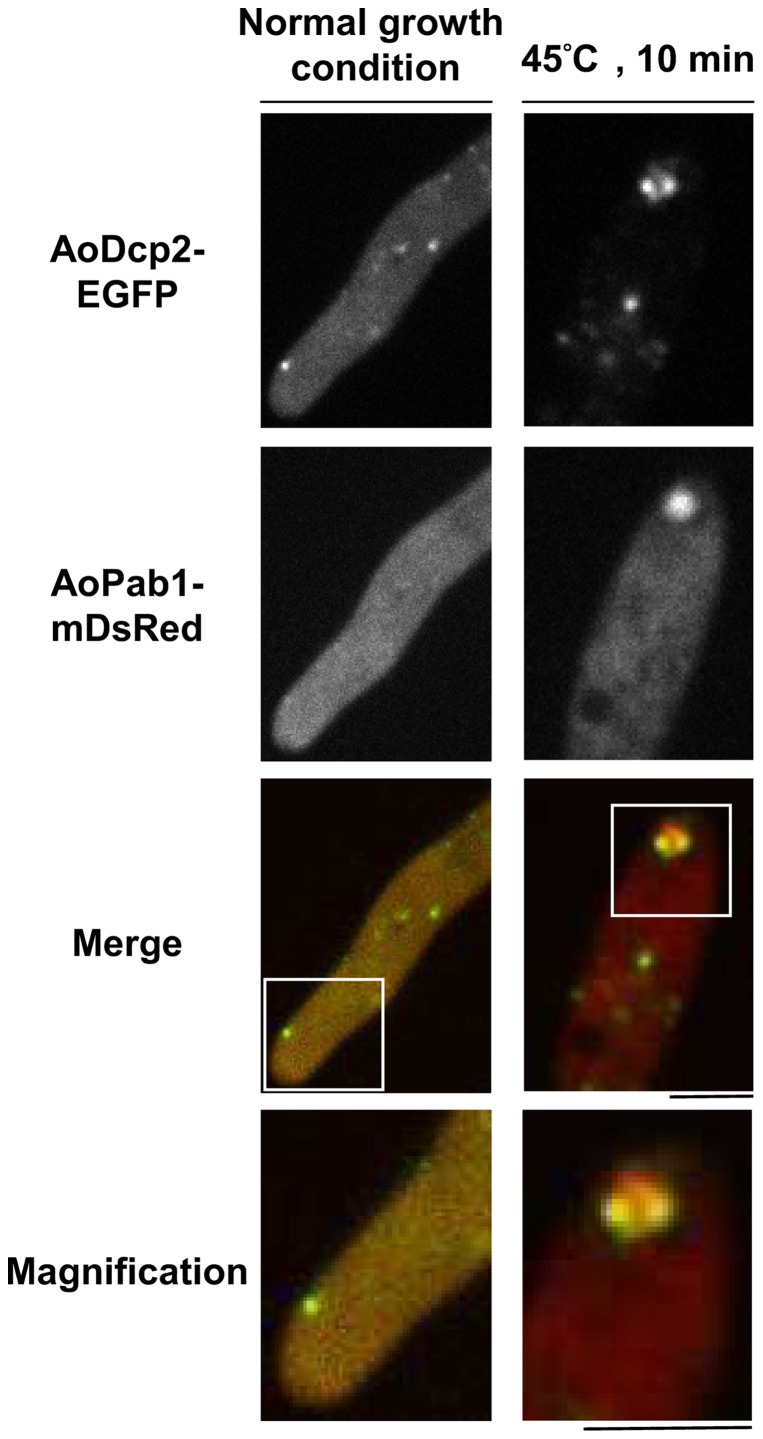
Subcellular localizations of P-bodies and stress granules in response to heat stress. AoDcp2-EGFP and AoPab1-mDsRed were used as markers of P-bodies and stress granules, respectively. Approximately 10^4^ conidia of cells co-expressing AoDcp2-EGFP and AoPab1-mDsRed were grown in CD medium at 30°C for 18 h before being exposed to 45°C for 10 min. The lowest panels show magnified images of the apical region of the cell (within the boxed area in the above image). Scale bars  =  5 µm.

In eukaryotic cells, non-translating mRNAs also accumulate in P-bodies, which contain a conserved core of proteins involved in translational repression and mRNA degradation. Although stress granules and P-bodies are compositionally and morphologically distinct entities, evidence suggests they are spatially and functionally linked [49], [51]–[55]. Dcp2p is the catalytic subunit of a decapping enzyme that cleaves the 5′cap of mRNA [56], [57] and is frequently found as a distinct component of P-bodies in *S. cerevisiae* [49]. To monitor P-bodies in *A. oryzae*, a homolog of *S. cerevisiae* Dcp2p, AoDcp2 (AO090120000363), was fused with EGFP and expressed under control of the *amyB* promoter. In unstressed cells, AoDcp2-EGFP was detected as discrete bright dots in the cytoplasm, in which it also showed a faint diffuse distribution (Figure S1). In *S. cerevisiae*, stress conditions such as glucose and nitrogen depletion, osmotic stress, and UV irradiation lead to an increase in the number of P-bodies [58], [59]. Here, we confirmed that P-bodies were increased in size and number when *A. oryzae* cells expressing AoDcp2-EGFP were exposed to heat stress, low temperature, and carbon deprivation (Figure S1). To determine the relative localizations of stress granules and P-bodies, an *A. oryzae* strain co-expressing AoDcp2-EGFP and AoPab1-mDsRed was examined after being exposed to 45°C for 10 min. Under heat stress, AoPab1-mDsRed cytoplasmic foci were colocalized with P-bodies labeled with AoDcp2-EGFP (Figure 3).

### AoSO colocalizes with stress granules upon heat stress

**Figure 4:**
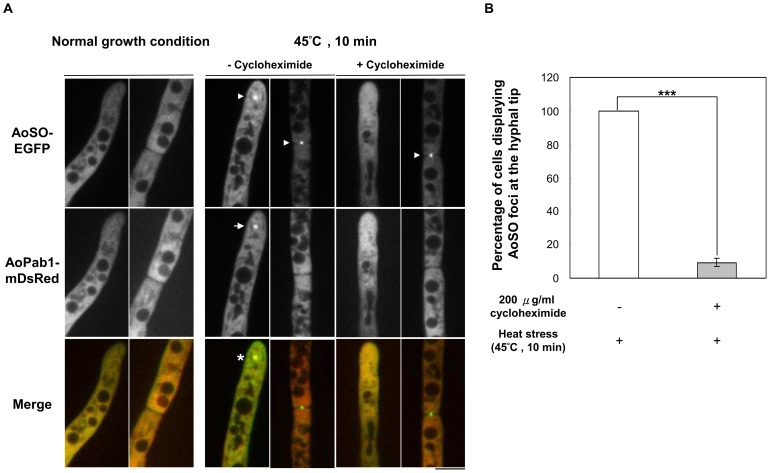
Subcellular localizations of AoSO-EGFP and AoPab1-mDsRed in response to heat stress. (A) A wild-type strain co-expressing AoSO-EGFP and AoPab1-mDsRed was used to examine the relative localizations of AoSO and stress granules under normal growth conditions and heat stress. Approximately 10^4^ conidia of cells were grown in CD medium at 30°C for 18 h before being exposed to 45°C for 10 min. Arrowheads indicate AoSO foci, and arrows indicate stress granules. Colocalization of a stress granule and an AoSO cytoplasmic focus is indicated by the asterisk. The effect of cycloheximide on the formation of mRNP granules was examined by pre-treating cells with 200 µg/ml cycloheximide for 30 min before being exposed to heat stress. Scale bar  =  5 µm. (B) Effect of cycloheximide on the formation of AoSO cytoplasmic foci at the hyphal tip. Cells were incubated with CD medium containing 200 µg/ml cycloheximide for 30 min before being exposed to heat stress. The percentage of cells displaying AoSO cytoplasmic foci at the hyphal tip was determined. Error bars represent the standard error. ***P < 0.0001. The presented data are from three independent experiments, each with n  =  50.

To investigate the possibility that AoSO protein is involved in the function of stress granules, a strain co-expressing AoSO-EGFP and AoPab1-mDsRed was used to examine the cellular localizations of AoSO and stress granules in cells exposed to heat stress (Figure 4A). The functionality of AoSO-EGFP was previously confirmed by demonstrating that expression of the fusion protein complemented the phenotypes of Δ*Aoso* strain [39]. Under normal growth conditions, AoSO-EGFP was evenly distributed throughout the cytoplasm, but accumulated at the septal pore after cells were exposed to heat stress, as previously reported [39]. However, AoPab1-mDsRed did not accumulate at the septal pore in cells exposed to heat stress (Figure 4A) or any other of the examined stress conditions (cold stress, glucose deprivation, and ER, osmotic and oxidative stresses; data not shown). In cells exposed to heat stress, AoSO-EGFP also accumulated as cytoplasmic foci, which colocalized with stress granules labeled with AoPab1-mDsRed at the hyphal tip (Figure 4A).

To further determine the physical association of AoSO with the stress granule component AoPab1, a strain co-expressing AoSO-EGFP and AoPab1-3HA was used in co-immunoprecipitation experiments. The interaction between AoSO-EGFP and AoPab1-3HA was confirmed by co-immunoprecipitation (Figure S2). This association is not mediated via the EGFP portion as no association was detected in the negative control strain co-expressing EGFP and AoPab1-3HA. However, the association between AoSO-EGFP and AoPab1-3HA was not induced or increased after cells were exposed to heat stress.

To clarify if the aggregation of AoSO requires the presence of non-translating mRNAs, cycloheximide, which blocks translational elongation and traps mRNAs in polysomes, was used to deplete the pool of non-translating mRNAs [49], [53], [60] (Figure 4A and 4B). The strain co-expressing AoSO-EGFP and AoPab1-mDsRed was treated with 200 µg/ml cycloheximide for 30 min before being exposed to heat stress. The formation of stress granules labeled with AoPab1-mDsRed was sensitive to the cycloheximide treatment, confirming they are typical mRNP granules, as previously reported [49], [53], [60]. In addition, the heat stress-induced formation of cytoplasmic AoSO foci at the hyphal tip was greatly impaired by cycloheximide (Figure 4A and 4B), suggesting that cytoplasmic AoSO foci require a pool of free mRNAs for their aggregation. However, cycloheximide did not affect the accumulation of AoSO at the septal pore. Overall, these results suggest that AoSO is a novel component of mRNP granules in the filamentous fungus *A. oryzae*.

### Deletion of *Aoso* affects the formation and localization of stress granules

**Figure 5:**
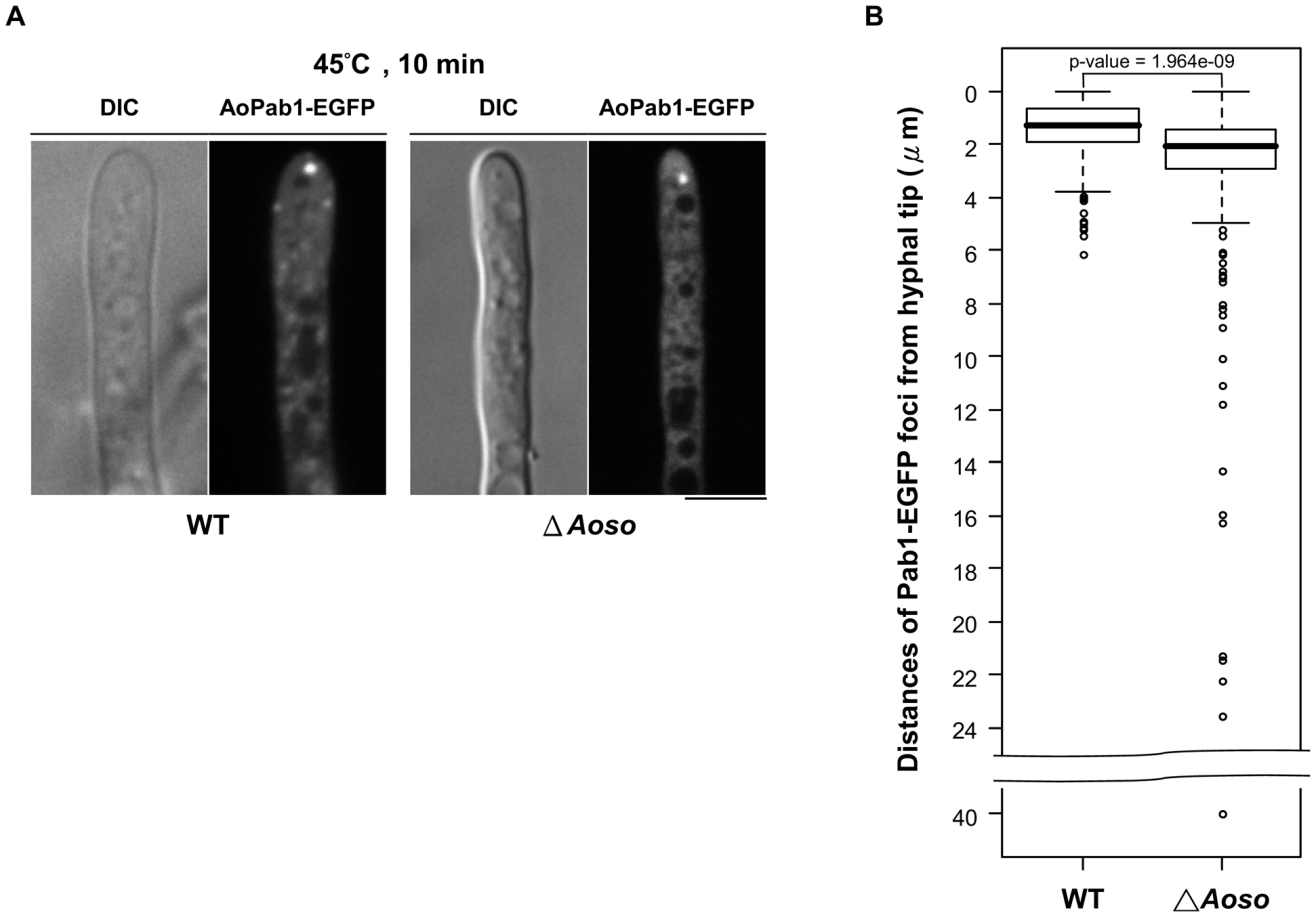
Effect of *Aoso* deletion on stress granules. (A) Stress granule formation in wild-type (WT) and *Aoso*-deletion mutant (▵*Aoso*) cells was detected using AoPab1-EGFP as a marker. Approximately 10^4^ conidia of cells expressing AoPab1-EGFP were grown in CD+Met medium at 30°C for 18 h before being exposed to 45°C for 10 min. Scale bar  =  5 µm. (B) Distribution of stress granule localization. The distance of AoPab1-EGFP foci from the hyphal tip is displayed using a box plot where the top and bottom of the box represent limits of the upper and lower quartiles, with the median being indicated by the horizontal line within the box. The whiskers show the highest and lowest reading within 1.5 times the interquartile range. The outliers are indicated by dots. The data were derived from three independent experiments with a total of 260 measurements in the WT and ▵*Aoso* strains, respectively.

**Figure 6:**
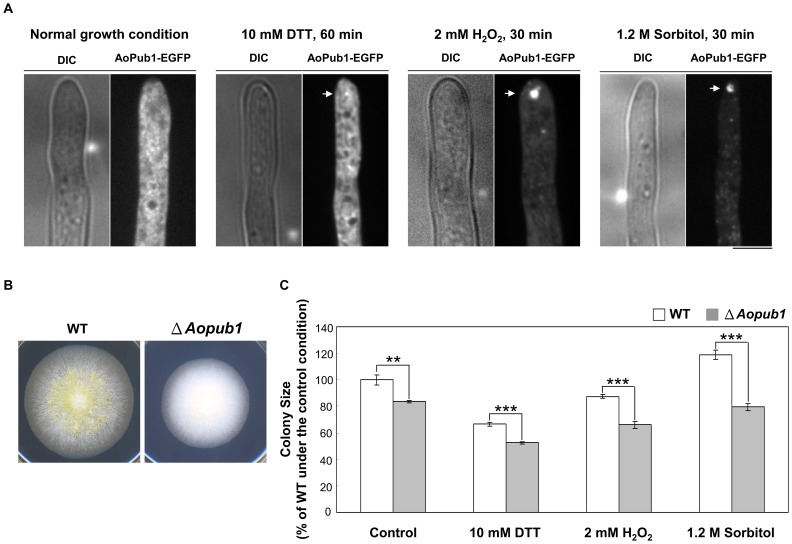
*Aopub1* disruptant showed defects in the conidia formation and more severe growth retardation in stress conditions. (A) Stress-induced formation of stress granules (indicated by arrows) in an AoPub1-EGFP expressing wild-type strain. Approximately 10^4^ conidia of cells were grown in CD+Met medium at 30°C for 18 h before being exposed to ER stress (10 mM DTT, 60 min), oxidative stress (2 mM H_2_O_2_, 30 min), and osmotic stress (1.2 M sorbitol, 30 min). Scale bar  =  5 µm. (B) Approximately 10^3^ conidia of wild-type and *Aopub1* disruptant cells were spotted onto PD plates, and cultured at 30°C for 4 days. (C) Approximately 10^3^ conidia of wild-type and *Aopub1* disruptant cells were spotted onto PD plates with or without 10 mM DTT, 2 mM H_2_O_2_, or 1.2 M sorbitol, and cultured at 30°C for 4 days. Colony diameters were compared to those of the wild-type strain under the control condition, which was set to 100%. Data represent the mean ± S.D. of three biological replicates. The asterisk denotes a statistically significant difference, as judged by the Student’s t-test with P < 0.005 (**) and P < 0.0005 (***).

To gain a better understanding of the role of AoSO in stress granules, the effect of *Aoso* deletion on stress granule formation was examined. Compared to 100% formation of stress granules in wild-type cells exposed to heat stress, the heat stress-induced formation of stress granules in the *Aoso*-deletion strain was decreased to 87.7 ± 1.34 % (hyphae = 50; n = 7; *p* < 0.005) (Figure 5A). No obvious change in the size of stress granules was observed in the *Aoso*-deletion strain. The movement of stress granules labeled with AoPab1-EGFP in stressed cells was monitored by live-cell imaging, which revealed that in contrast to other types of stress where the stress granules were highly dynamic (Video S2 for oxidative stress and data not shown), the heat stress-induced stress granules were nearly stationary (Video S3). By taking advantage of this feature, the effect of *Aoso* deletion on stress granules was further evaluated by measuring the distance between the hyphal tip and stress granules. We found that the distribution of the largest stress granules labeled with AoPab1-EGFP was less concentrated and more distant from the hyphal tip in the *Aoso*-deletion strain (Figure 5B). Additionally, we observed that in a small portion of hyphae, heat stress-induced stress granules were more dynamic in the *Aoso*-deletion strain (Video S4). However, the motility of heat-stress induced stress granules in the *Aoso*-deletion strain was different from that of all the other stress conditions examined in this study with a long distance movement (Video S2), but moved around in a confined region (Video S4). We have examined the growth test of the *Aoso* deletion strain in the stress conditions used in Figure 6; however, no growth difference between wild-type and the *Aoso* deletion strain was observed. Taken together, the results indicated that AoSO is not absolutely necessary for the formation of stress granules, but AoSO influences the formation and localization of stress granules in cells exposed to heat stress.

### *Aopub1* disruptant has defects in the conidia formation and displays more severe growth retardation in stress conditions

The ability to form stress granules is correlated with the survival of cells exposed to stress [5]. The C-terminal regions of TIA-1 and TIAR (mammalian homologs of AoPub1), and their orthologs, contain a glutamine/asparagine (Q/N)-rich prion-like domain, which have a strong tendency to self-aggregate and promote stress granule formation [60]–[63]. To investigate whether the formation of stress granules influences cell survival against stress, an *Aopub1* disruptant was constructed and cultured on PD plates containing 10 mM DTT, 2 mM H_2_O_2_, or 1.2 M sorbitol to examine sensitivity to these three types of stress. The induction of stress granules was confirmed in the AoPub1-EGFP expressing strain under each of the stress conditions (Figure 6A). The phenotype of the *Aopub1* disruptant was characterized by a slower growth rate compared to wild-type cells, and a severe impairment in the formation of conidia (Figure 6B). The growth retardation of the *Aopub1* disruptant was more severe under all examined stress conditions (Figure 6C). Colony sizes of *Aopub1* disruptant compared with those of wild-type strain were reduced by 16.5%, 21.1%, 24.7%, and 33.2% in the control, 10 mM DTT, 2 mM H_2_O_2_, and 1.2 M sorbitol conditions, respectively.

## Discussion

### Stress granules are conserved in *A. oryzae*

Stress granules are widely observed in eukaryotes; however, prior to the present study, these mRNP granules had not yet been defined in filamentous fungi. We used EGFP-fused AoPab1 as a stress granule marker, and observed that AoPab1 accumulated as cytoplasmic foci at the hyphal tip of cells exposed to various stresses. This finding indicates that the formation of stress granules is a general phenomenon in response to external stress. To our knowledge, the present study is the first to identify stress granules in a filamentous fungus, which suggests that stress-induced reprogramming of mRNAs to enter stress granules occurs in *A. oryzae*.

Although P-bodies and stress granules are compositionally and functionally distinct structures, accumulating evidence suggests they are spatially and functionally associated [49], [51]–[55]. Here, we showed that AoPab1-mDsRed, which is the core component of stress granules, colocalizes with P-bodies labeled with AoDcp2-EGFP. The spatial relation of stress granules and P-bodies is also seen in mammalian cells [51] and *S. cerevisiae* [49], [53]. The stress-induced formation of stress granules, in which mRNAs are not degraded, but maintained in a nontranslating state, is thought to make mRNAs available for rapid reinitiation when cells recover from stress. In agreement with this speculation, stress-induced stabilization of liable mRNAs has been reported [64]–[66], suggesting that certain components of the mRNA degradation pathway are impaired in response to stress. This finding may explain why the number and size of P-bodies were increased coordinately in *A. oryzae* cells under stress (Figure 3 and Figure S1).

It is noteworthy that in mammalian and yeast cells, numerous stress granules are distributed throughout the cytoplasm following exposure to stress; however, only a few stress granules that were located predominately at the hyphal tip were found in *A. oryzae*. Although the underlying reason for this observation is not yet clear, the polarized localization of stress granules suggests a spatial specificity of the posttranscriptional regulation of gene expression in *A. oryzae*. It is well known that filamentous fungi have a highly polarized cell structure in which secretory vesicles, cytoskeletal elements, and related components are concentrated at the hyphal tip as a well-organized cluster that determines hyphal growth and polarity [67], [68]. The localization of ribosomes at the hyphal tip supports the idea that mRNA translation actively occurs in this region [69]. Based on these findings, we speculate that asymmetrical localization of mRNA at the hyphal tip results in the spatially restricted formation of stress granules or that mRNA at the hyphal tip is preferentially routed into stress granules as an acute response to stress.

### AoSO is a component of stress granules

Deletion of the *so* gene in *N. crassa* results in a pleiotropic phenotype characterized by a lack of hyphal anastomoses, reduced aerial hyphae, slower growth rate, altered conidiation pattern, and female sterility [37]. Mutation of the conserved WW domain in SO, which is predicted to mediate protein-protein interactions, does not affect SO localization to the septal pore; however, the phenotypic defects of the *so* disruptant are not fully complemented [35]. Clearly, SO is a multi-function protein, and a plugging of the septal pore is not sufficient to explain the multiple phenotypic defects observed in the *so*-deletion strain. The molecular function of the SO protein remains largely unknown. Localization analysis has revealed that the *N. crassa* SO homolog *S. macrospora* Pro40 partially associates with Woronin bodies [38], and that *N. crassa* SO contributes to the sealing efficiency of pores plugged by Woronin bodies after hyphal injury [35]. Additionally, cell-cell signaling and tropic growth of *N. crassa* germlings involve the unusual subcellular dynamics of SO and the MAP kinase (MAK-2), which are recruited to the plasma membrane of cell tips of interacting germlings in an oscillating and alternating manner [36]. In the present study, we found that AoSO accumulates not only at the septal pore, but also at the hyphal tip, in cells exposed to heat stress. In addition, cytoplasmic AoSO foci colocalized with AoPab1-mDsRed-labeled stress granules at the hyphal tip and were sensitive to cycloheximide treatment (Figure 4), suggesting that cytoplasmic AoSO foci are mRNP granules, and that AoSO therefore may participate in the posttranscriptional regulation of mRNA in response to heat stress. The physical association between AoSO-EGFP and AoPab1-3HA was further confirmed by co-immunoprecipitation (Figure S2); however, this association was not induced or increased after cells were exposed to heat stress. An inconsistence between the results of co-immunoprecipitation and colocalization analysis may be explained by the different culture conditions (DPY complete medium in submerged culture and CD+Met minimal medium in stationary culture). Orthologs of the *so* gene have only been identified in the genomes of Pezizomycotina species [37], therefore we presume that AoSO is a novel component of stress granules specific to Pezizomycotina. The effect of *Aoso* deletion on the function of stress granules revealed that AoSO is not indispensable for stress granule formation; however, the formation and localization of stress granules were influenced in the absence of AoSO (Figure 5).

Protein-protein interaction has been implicated in the assembly of mRNP granules, including stress granules. One mechanism of assembly is mediated through the glutamine/asparagine (Q/N)-rich prion-like domain, which has a strong tendency to self-aggregate and is found in many components of mRNP granules, including TIA-1 and TIAR (mammalian homologs of AoPub1) [60]–[63]. Moreover, the long, intrinsically disordered domains identified in septal pore-associated (SPA) proteins show an inherent tendency to aggregate [70]. The N-terminal domain of AoSO is also predicted to be disordered (data not shown), suggesting that it has the potential to form aggregates of mRNP granules, although *N. crassa* SO, which contains a disordered domain that is enriched in glutamine, fails to form aggregates *in vitro* [70]. In consistence with this property, deletion of *Aoso* resulted in an 12.3 ± 1.34 % reduction in the number of tip cells displaying stress granules labeled with AoPab1-EGFP under the heat stress condition. We also observed that in a small portion of hyphae, heat stress-induced stress granules were more dynamic in the *Aoso*-deletion strain (Video S4). It remains unclear how AoSO influences the localization and motility of stress granules. AoSO contains a conserved WW domain, as well as a proline-rich domain predicted to mediate protein-protein interactions. The localization and dynamics of stress granules may be indirectly influenced through protein-protein interactions of AoSO with other proteins (Figure 5 and Video S4). Our present findings, in addition to the known functions of AoSO and its homologs in hyphal fusion, sexual reproduction, and septal plugging [37]–[39], raise the possibility that AoSO participates in the posttranscriptional regulation of mRNA in response to heat stress. However, the localization of mRNAs in AoSO cytoplasmic foci remains to be conclusively demonstrated.

### *Aopub1* disruptant has defects in the conidia formation and displays more severe growth retardation in stress conditions

The formation of stress granules in response to stress is widely observed in eukaryotes. Although the physiological role of the assembly of mRNAs into these large aggregate-like assemblies remains unclear, this evolutionarily conserved response suggests that stress granules serve important functions in eukaryotes. Although relatively little is known about the mechanisms regulating the formation of stress granules, their assembly seems to be mediated through a self-assembly process that involves the Q/N-rich prion-like domains of a number of protein components associated with stress granules, including TIA-1, a mammalian homolog of AoPub1 [7], [61], [62], [71], [72]. As Pab1p is an essential protein in *S. cerevisiae* and is not required for stress granule formation [73], we investigated whether deletion of the *Aopub1* gene confers cellular sensitivity to stress. The *Aopub1* disruptant displayed a slower growth rate compared to wild-type cells, and the growth retardation was more severe when cells were cultured under stress conditions, which included ER, oxidative, and osmotic stresses. Although we lack direct evidence that the formation of stress granules is impaired in the *Aopub1* disruptant, a Δ*pub1* strain of *S. cerevisiae* displays a dramatic decrease in the average number of stress granules, as judged by Pab1p localization [49], [74]. The data presented here suggest that the integrity of stress granules is important for the survival of *A. oryzae* cells exposed to stress.

## Supporting Information

Figure S1 **P-bodies under normal growth condition and in response to stresses.** Approximately 10^4^ conidia of cells expressing AoDcp2-EGFP were grown in CD+Met medium at 30°C for 18 h before being exposed to various stresses. For temperature stress, cells were shifted from 30°C to 4°C for 30 min or to 45°C for 10 min. For glucose deprivation, cells were washed three times with CD medium without glucose, and further incubated for 10 min in CD medium without glucose. Scale bar  =  5 µm.
(TIF)

Figure S2 **Co-immunoprecipitation of AoSO-EGFP and AoPab1-3HA.** (A) Approximately 10^7^ conidia of cells expressing AoSO-EGFP and AoPab1-3HA or EGFP and AoPab1-3HA were inoculated in 20 ml DPY medium (2% dextrin, 1% polypeptone, 0.5% yeast extract, 0.5% KH_2_PO_4_, 0.05% MgSO_4_·7H_2_O), and cultured at 30°C for 10 h before being exposed to heat stress. Mycelia were harvested by filtration, frozen in liquid nitrogen, and pulverized using a multibead shocker (Yasui kikai, Osaka, Japan). Proteins were extracted in lysis buffer (10 mM Tris-HCl [pH 7.5], 150 mM NaCl, 0.5% NP-40, 0.5 mM EDTA, 1 mM PMSF, and 1x protease inhibitor cocktail [Sigma, St.Louis, MO, USA]). After centrifugation at 5,000 × g for 10 min, the supernatants were incubated with anti-HA-tag mAb-Magnetic Agarose beads (Medical & Biological Laboratories Co. Ltd., Japan) for 2 hr. Immune complexes were washed four times with wash buffer (10 mM Tris-HCl [pH 7.5], 300 mM NaCl, 0.5% NP-40, 0.5 mM EDTA, 1 mM PMSF), and subjected to immunoblotting. Anti-GFP antibody (1∶3,000 dilution; Funakoshi Co. Ltd.) or anti-HA (12CA5) (1∶1,000 dilution; Roche Molecular Biochemicals) antibody was used as primary antibody. Anti-mouse immunoglobulin G labeled with peroxidase (1∶1,000 dilution; Funakoshi Co. Ltd, Tokyo, Japan) was used as secondary antibody. (B) A strain expressing AoSO-EGFP alone was used as a negative control (right lane). Co-immunoprecipitation was performed as described in (A).
(TIF)

Video S1 **Time-lapse capture of the subcellular movements of AoPab1-EGFP revealed the fusion of stress granules.** Approximately 10^4^ conidia of AoPab1-EGFP expressing cell were grown in CD+Met medium at 30°C for 18 h before being exposed to oxidative stress (2 mM H_2_O_2_). Single focal planes were captured at 550 ms intervals (total 32 frames). The video is presented at 5 frames/s.
(AVI)

Video S2 **Time-lapse capture of the subcellular movements of AoPab1-EGFP in the wild-type strain after cells were exposed to oxidative stress for 10 min.** Note that the stress granule labeled with AoPab1-EGFP at the hyphal tip moved toward to the subapical region with a long distance. Cells were cultured and examined under the same condition described in the Video S1. Single focal planes were captured at 550 ms intervals (total 100 frames). The video is presented at 5 frames/s.
(AVI)

Video S3 **Time-lapse capture of the subcellular movements of AoPab1-EGFP in the wild-type strain revealed that the heat stress-induced stress granule labeled with AoPab1-EGFP at the hyphal tip was nearly stationary.** Approximately 10^4^ conidia of cell were grown in CD+Met medium at 30°C for 18 h before being exposed to heat stress. Single focal planes were captured at 550 ms intervals (total 100 frames). The video is presented at 5 frames/s.
(AVI)

Video S4 **Time-lapse capture of the subcellular movements of AoPab1-EGFP in the Δ** ***Aoso*** **stain revealed that a confined movement of the heat stress-induced stress granule.** Note the stress granule labeled with AoPab1-EGFP at the subapical region. Cells were cultured and examined under the same condition described in the Video S3. Single focal planes were captured at 550 ms intervals (total 100 frames). The video is presented at 5 frames/s.
(AVI)
