# Supplementary figures and images for: Aspergillus oryzae AoSO Is a Novel Component of Stress Granules upon Heat Stress in Filamentous Fungi
Source: PLoS One. 2013 Aug 21;8(8):e72209. doi: 10.1371/journal.pone.0072209 (PMC3749109; doi:10.1371/journal.pone.0072209)

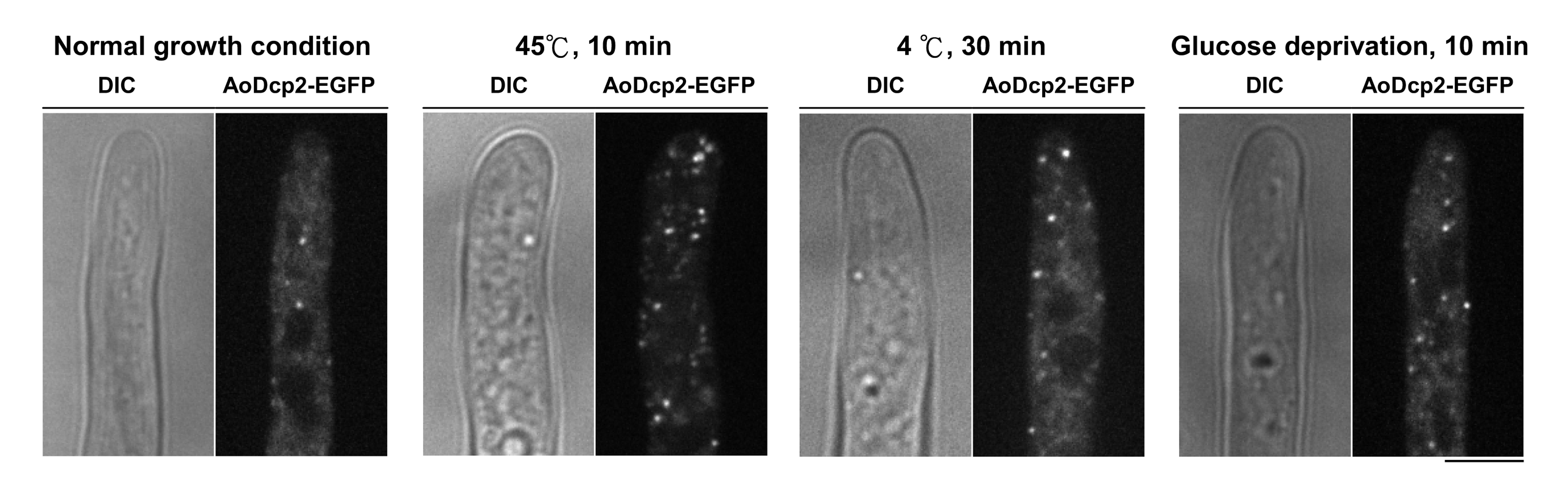

Supplement: Figure S1 — P-bodies under normal growth condition and in response to stresses. Approximately 104 conidia of cells expressing AoDcp2-EGFP were grown in CD+Met medium at 30°C for 18 h before being exposed to various stresses. For temperature stress, cells were shifted from 30°C to 4°C for 30 min or to 45°C for 10 min. For glucose deprivation, cells were washed three times with CD medium without glucose, and further incubated for 10 min in CD medium without glucose. Scale bar = 5 µm. (TIF) [file pone.0072209.s001.tif]
